# Placental Transmission of Human Parvovirus 4 in Newborns with Hydrops, Taiwan

**DOI:** 10.3201/eid1710.101841

**Published:** 2011-10

**Authors:** Mao-Yuan Chen, Shiu-Ju Yang, Chien-Ching Hung

**Affiliations:** National Taiwan University Hospital, Taipei, Taiwan

**Keywords:** viruses, parvovirus B19V, parvovirus 4, hydrops, vertical transmission, infants, dispatch

## Abstract

In studying the epidemiology of parvovirus 4 (PARV4) in Taiwan, we detected DNA in plasma of 3 mothers and their newborns with hydrops. In 1 additional case, only the mother had PARV4 DNA. Our findings demonstrate that PARV4 can be transmitted through the placenta.

Transmission routes of human parvovirus 4 (PARV4), a recently discovered member of the *Parvoviridae* family (*1*), are not fully understood; studies have suggested that PARV4 is transmitted predominantly through the parenteral route (*2**,**3*). To study the epidemiology of PARV4 infection in Taiwan, we developed an in-house PARV4 immunoblot (*4*). During the process, we found regions of higher similarity in amino acid sequence between PARV4 and parvovirus B19 virus (B19V). They are LPG***T***NYVGPGN***E***L (B19V VP1, aa 125–137) LPG***Y***NYVGPGN***P***L (PARV4 open reading frame [ORF] 2, aa 219–231) and Y***K***YPYVLG***QG***QDTL (B19V VP2, aa 157–170) Y***D***YPYVLG***HN***QDTL (PARV4 ORF2, aa 499–512).

To exclude the possibility of antibody cross-reaction between PARV4 and B19V, we tested plasma samples sent to our laboratory for confirmation of B19V infection with PARV4 immunoblot. Unexpectedly, we detected PARV4 DNA in plasma from a mother and her newborn with hydrops. Therefore, we examined samples from 5 additional infants with hydrops.

## The Study

During 2000–2009, our laboratory received blood samples from 6 infants with nonimmune idiopathic hydrops (Table 1). Paired mother–newborn plasma samples from 4 infants were available for this study; plasma from either the mother or newborn was missing in 2 instances. None of the blood samples from the newborns was cord blood. All infants (case-patients) had at least 2 of the following conditions: ascites, pleural effusion, pericardial effusion, skin edema, or polyhydramnios.

**Table 1:** Clinical information about 6 infants with hydrops, Taiwan, 2000–2009*

Patient	Sex	Delivery	Gestational age, wk	Birthweight, g	Hydrops signs	Hemoglobin, g/dL	Transfusion†	Platelets, 10^3^/μL	Outcome
A	F	CS	35	2,846	Pericardial effusion, polyhydramnios	8.1	Yes	NA	Survived
B	M	Vaginal	40	2,468	Pleural effusion, skin edema	7.9	Yes	183	Survived
C	F	CS	32	3,070	Pleural effusion, skin edema	7.8	Yes	22	Died
D	M	CS	35	3,030	Ascites, pericardial effusion	4.1	Yes	6	Survived
E	M	Vaginal	27	1,450	Pleural effusion, skin edema	13	No	232	Survived
F	M	CS	32	2,634	Ascites, pleural effusion	12	No	76	Died

*CS, cesarean section; NA, not available. †All transfusions were given after delivery. Blood samples were collected on the second (patients A–D) or third (E, F) day after delivery.

Antibodies to PARV4 and B19V were tested by immunoblots. DNA of PARV4 and B19V was detected by seminested and nested PCR, respectively. PARV4 immunoblot and PCR were performed according to the methods in our previous report (*4*). The B19V immunoblot and PCR are described in the Technical Appendix. The 2 fragments of the PARV4 capsid protein, aa 272–630 and aa 604–914 of ORF2, were fused to bacterial small ubiquitin-like modifier (SUMO) protein (a member of a ubiquitin-like protein family) and used as antigens in immunoblot. They were named viral protein (VP) 2 and VP3. For B19V, the antigens were VP1-specific (VP1-S; VP1, aa 1–227) fused to thioredoxin and VP2N (N terminal of B19V VP2, aa 1–343) fused to SUMO. The control protein was ribosomal P2 protein fused to SUMO. Antibodies to ribosomal P2 protein were rarely detected, except in patients with systemic lupus erythematosus (*5*).

Four of the 5 mothers had immunoglobulin (Ig) M against PARV4 (Table 2). Two of the 4 also had IgG against PARV4 (Figure, A, E); the other 2 had weakly positive IgM without IgG (not shown). No newborn had IgM against PARV4. We detected IgM against B19V in only 1 mother (Figure, A), who also had IgM against PARV4. None of the newborns had IgM against B19V. Two mothers (Figure, B, E) and 2 newborns had IgG against VP2N but not VP1S. The immunoblot pattern of IgG against B19V was inconsistent with findings in a previous report (*6*).

**Table 2:** Antibody to B19V and PARV4 and detection of viral DNA in mothers and newborns, Taiwan, 2000–2009*

Case and patient	B19V			PARV4	
	Antibody	DNA		Antibody	DNA
A					
Mother	IgM+, IgG+; anti-VP1 and 2	+		IgM+, IgG+	+
Newborn	IgM–, IgG+; anti-VP1 and 2	+		IgM–, IgG+	+
B					
Mother	IgM–, IgG+ to VP2 only	–		IgM–, IgG+	+
Newborn	IgM–, IgG+; to VP2 only	–		IgM–, IgG+	+
C					
Mother	IgM–, IgG–	–		IgM weak +, IgG–	+
Newborn	IgM–, IgG–	–		IgM–, IgG–	–
D					
Mother	IgM–, IgG–	–		IgM weakly positive, IgG–	+
Newborn	IgM–, IgG–	–		IgM–, IgG–	+
E					
Mother	IgM weakly positive, IgG+, to VP2 only	–		IgM+, IgG+	+
F					
Newborn	IgM–, IgG+; to VP2 only	–		IgM–, IgG+	+

*B19V, parvovirus B19 virus; PARV4, parvovirus 4; Ig, immunoglobulin; +, positive; VP, viral protein; –, negative.

**Figure:**
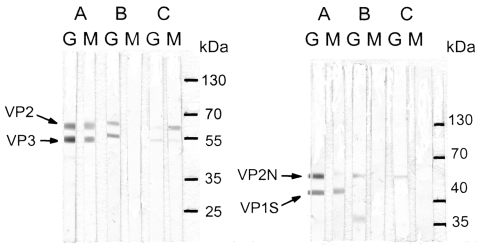
Immunoglobulin (Ig) G and IgM immunoblots of 3 mothers for infection with parvovirus 4 (PARV4) (left) or parvovirus B19 (B19V) (right). Case-patient A was co-infected with PARV4 and B19V; case-patient B was the only mother who did not have IgM against PARV4; case-patient E had weak IgM against PARV4 viral protein (VP) 3 and IgM against B19V VP2N, which could not be seen after scanning. Molecular weights are ≈60 kDa for PARV4 VP2, 51 kDa for PARV4 VP3, 51 kDa for B19V VP2N, and 41 kDa for B19V VP1S.

Only the mother and newborn of case A had detectable B19V DNA (genotype 1). By contrast, PARV4 DNA (genotype 2) was found in plasma of all but 1 of the 6 case-patients. The newborn negative for PARV4 DNA received a whole-blood exchange before sampling.

## Conclusions

The first serologic study (*7*) and a recent study (*8*), both conducted in northern Europe, supported the fact that PARV4 is primarily a blood-borne virus. PARV4 DNA was detected in blood donors (*9**,**10*), and detection rates were 2% and 3.95%, respectively. The PARV4 seropositivity rate is expected to be higher than the DNA detection rate in blood donors because of the possibility of past infection (*2**,**11**,**12*). However, the IgG seroprevalence in 199 blood donors in France was 0%; the same rate was found in the general population in the United Kingdom (*13*). A much lower PARV4 DNA detection rate in blood donors in France may explain the result. Inconsistent with the findings of extremely low seroprevalence in France and the United Kingdom, PARV4 DNA was detected in the liver (15% and 41%, respectively) and the heart (41%) of non–HIV-infected patients in Germany (*11*) and Italy (*14*). PARV4 infection might be more widespread in some countries in Europe.

Contrary to the epidemiology of PARV4 in Europe, studies in Africa found different transmission routes and a higher seropositive rate in blood donors and the general population. In Ghana, 8.6% of infants had PARV4 viremia (*15*). In sub-Saharan Africa, 20%–37% of adults studied had antibodies to PARV4 (*13*). The groups studied in both reports did not have parenteral risk.

PARV4 can be transmitted through nonparenteral routes (*13**,**15*). Our study showed that placental transmission is one of them. PARV4 was unlikely to have been transmitted through a blood transfusion because of the low detection rate of PARV4 DNA in the blood donors. Because Taiwan has a high PARV4 seroprevalence rate (*4*), the possibility of a higher PARV4 DNA detection rate in blood donors is of concern. However, considering that the PARV4 seropositivity rate was 76.8% in HIV-infected intravenous drug users but only 6 of 350 had detectable DNA (*14*), the concern is not realistic.

Maternal PARV4 infections were diagnosed by detection of PARV4 DNA in all 5 mothers; 4 of whom had IgM against PARV4. Using IgM against PARV4 as evidence of recent infection must be done cautiously because of persistent IgM against PARV4 (*4*). Two mothers had weak IgM but no IgG against PARV4. The possibility of nonspecific IgM binding is low because of PARV4 viremia. The IgM result may be negative if the 2 samples are tested by enzyme immunoassay. The 2 mothers might have defective humoral immunity against PARV4 because we had detected 4 non–HIV-infected patients who had persistent IgM against PARV4 but did not have (or had weakly positive) IgG against PARV4 over 9–35 months. In a mother without IgM against PARV4, the amount of IgM might rapidly decline or a relapse of viremia might occur. In our previous longitudinal study of blood with IgM against PARV4, we found PARV4 DNA transiently during follow-up in 1 case (*4*). The mother without IgM against PARV4 was pregnant again 2 years later, and fetal death occurred at 18 weeks’ gestation.

Persons with past B19V infection are expected to have IgG against B19V VP1 but not VP2 in immunoblot (*6*). On the contrary, 4 samples in this study had IgG against VP2N but not VP1S. We excluded the possibility of a reaction with SUMO protein by testing with the control protein. We tested 32 samples that had IgG against PARV4 and B19V VP2N using a commercial IgG B19V enzyme immunoassay (IBL, Hamburg Germany); 9 tested positive by IBL, and 8 were definitely positive because IgG against VP2N and VP1S were positive in our B19V immunoblot. Twenty-four samples had IgG against VP2N but not VP1S, only one of which tested positive by IBL. The paradoxical result was not seen in 47 blood samples without IgG against PARV4. Therefore, the best explanation is that PARV4 antibodies can cross-react with those of B19V VP2N.

In conclusion, PARV4 can be transmitted parenterally and placentally. Other transmission routes might exist and remain to be discovered. Prospective studies of PARV4 infection during pregnancy are needed to clarify the effect of PARV4 infection on fetal outcome.

## Supplementary Material

Technical Appendix Infants with nonimmune idiopathic hydrops fetalis are not routinely checked for parvovirus B19 infection.
