# Supplementary material for: Placental Transmission of Human Parvovirus 4 in Newborns with Hydrops, Taiwan
Source: Emerg Infect Dis. 2011 Oct;17(10):1954–6. doi: 10.3201/eid1710.101841 (PMC3310659; doi:10.3201/eid1710.101841)
Supplement: Technical Appendix — Infants with nonimmune idiopathic hydrops fetalis are not routinely checked for parvovirus B19 infection. [file 10-1841-Techapp_2p.pdf]

Article DOI: http://dx.doi.org/10.3201/eid1710.101841

Placental Transmission of Human Parvovirus 4 in Newborns with Hydrops Fetalis, Taiwan

Technical Appendix

Blood Samples

Infants with nonimmune idiopathic hydrops fetalis are not routinely checked for parvovirus B19 infection. That is why only 6 cases were available for the present study. Other causes of hydrops had been excluded, such as genetic causes and cardiovascular abnormalities. None of the 6 mothers was an injection drug user.

Methods

B19 Recombinant Fusion Proteins

Two B19 proteins were made, viral protein VP1S (VP1-specific, VP1 aa 1–227) and VP2N (N-terminal of B19 VP2, aa 1–343). VP1S fragment was produced by PCR by using plasma B19 DNA from HIV-1–infected patient CH (*1*) as template. The primers used were VP1S sense 5′-CTCGAGATGAGTAAAGAAAGTGGCAA (nt 2444–2463, number according to Au isolate, Xho I restriction enzyme site at 5′), VP1S antisense 5′-GAATTCGCTTGGGTATTTTTCTGAGGC (nt 3124–3104, EcoRI enzyme site at 5′), VP2N sense (nt 3125–3144) 5′-ATGACTTCAGTTAATTCTGC and VP2N antisense (nt 4159–4136) 5′-TTATTTATCTGTGTCCCAGTGGTG. The cycling conditions were: 94oC for 2 min, followed by 35 cycles of 94oC for 30 sec, 55 oC for 30 seconds, 72 oC for 2 min, and a final extension of 7 min at 72 oC. VP1S PCR product was cloned by TA cloning kit (pCRII Invitrogen, Carlsbad, CA, USA). The cloned VP1S gene was cleaved out by restriction enzyme Eco RI and Xho I and then ligated to pThioHis A protein expressing vector (Invitrogen). The VP2N PCR product was ligated to pET SUMO vector (Invitrogen). The production and purification of recombinant fusion proteins were done according to manufacturer’s manuals.

B19 Immunoblot

The immunoblot method was the same as PARV4 immunoblot (*2*).

B19 PCR

DNA was extracted from 200 µL plasma by using High Pure Viral Nucleic Acid Kit (Roche, Mannheim, Germany). The final elution volume was 50 µl, and 5 µL was used in the reaction. B19 DNA was detected by nested PCR. The primers were P1f, P1r, n-P1f and n-P1r (*3*). The cycling conditions were: 94oC for 2 minute, followed by 35 cycles of 94oC for 30 sec, 55 oC for 30 sec, 72 oC for 30 sec, and a final extension of 2 min at 72 oC. The PCR products were purified by QIAquick PCR purification kit (QIAGEN) and sequenced by DyeDeoxy Terminator Cycle Sequencing Kit (Applied Biosystems).

References

1. Hung CC, Sheng WH, Lee KL, Yang SJ, Chen MY. Genetic drift of parvovirus B19 is found in AIDS patients with persistent B19 infection. J Med Virol. 2006;78:1374–84. PubMed doi:10.1002/jmv.20708

2. Yang SJ, Hung CC, Chang SY, Lee KL, Chen MY. Immunoglobulin G and M antibodies to human parvovirus 4 (PARV4) are frequently detected in patients with HIV-1 Infection. J Clin Virol. 2011;51:64–7. PubMed doi:10.1016/j.jcv.2011.01.017

3. Toan NL, Duechting A, Kremsner PG, Song LH, Ebinger M, Aberle S, et al. Phylogenetic analysis of human parvovirus B19, indicating two subgroups of genotype 1 in Vietnamese patients. J Gen Virol. 2006;87:2941–9. PubMed doi:10.1099/vir.0.82037-0
